# Animal‐Free Derived Collagen‐Like Protein Based Electrospun Nanofibers for Biomedical Applications: Cell Interactions Studies

**DOI:** 10.1002/mabi.70180

**Published:** 2026-04-09

**Authors:** Christoph Krauss, Maria Montero Mirabet, Blerta Sonja Thaqi, Sven Weber, Karsten Mäder

**Affiliations:** ^1^ Institute of Pharmacy Martin Luther University Halle‐Wittenberg Halle (Saale) Germany; ^2^ Evonik Operations GmbH; Research, Development & Innovation Darmstadt Germany

**Keywords:** biocompatibility, biomaterial, crosslinking, collagen‐like protein, drug delivery, electrospinning, tissue engineering

## Abstract

Biocompatibility of biomaterials is essential for their application in the biomedical field, particularly in tissue engineering and drug delivery systems. This study focuses on VECOLLAN, a recombinant, non‐animal‐derived collagen‐like protein (CLP), which exhibits excellent biocompatibility while promoting in vitro cell proliferation and wound healing. We previously optimized electrospinning parameters and employed a coaxial crosslinking approach to produce VECOLLAN‐based fibers with tunable dissolution, swelling behavior, and elasticity suitable for biomedical applications. The current study aims to assess the compatibility of these fibers with cells (NIH/3T3), investigate chemical leachables from three different formulations of DMTMM cross‐linked VECOLLAN‐fibers (according to ISO 10993‐18), and conduct spectroscopic analysis to confirm crosslinking efficacy. Results indicate that most CLP‐based nonwoven mats maintained cell viability above the 70% safety threshold as per ISO‐10993‐5. The sample with a CLP:DMTMM ratio of 1:0.1 demonstrated the most favorable cell compatibility and effective crosslinking. While the coaxial crosslinking method showed efficiency, residual crosslinker molecules and unexpected derivatives are identified. Spectroscopic investigations gave hints of successful crosslinking, although a direct correlation between crosslinker concentration and spectral band intensity is not established. Future research shall explore additional crosslinkers and cell types to further investigate the biocompatibility and potential applications of VECOLLAN‐based nonwoven mats.

## Graphical abstract

This study evaluates the biocompatibility of VECOLLAN, a recombinant collagen‐like protein, for biomedical applications. Using optimized electrospinning and coaxial crosslinking, fibers with tunable properties are produced. Cell adhesion properties and compatibility are assessed, revealing promising results for safety and functionality. Future work will explore additional crosslinkers and cell types to enhance the potential of VECOLLAN‐based nonwoven mats.

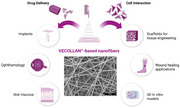

## 1 Introduction

The compatibility and safety of biomaterials play a crucial role in biomedical applications, particularly in tissue engineering and drug delivery systems [1]. Biomaterials should be safe, avoiding harmful effects such as cytotoxicity, genotoxicity, or immunogenicity, while fulfilling their intended function and promoting a favorable cellular or tissue response [2, 3]. Besides the potential toxicity of a material itself, potential degradation during storage or at the application site has to be evaluated carefully [4, 5]. Especially, synthetic biomaterials are attractive because they enable precise control over structure, composition, and functionality [6].

Biomaterials often lack the necessary mechanical properties and stability in aqueous environments for use in the biomedical sector. Combining crosslinkers with biomaterials is essential for creating effective, personalized, and sustainable medical solutions. However, many crosslinkers either adversely affect the functionality of biopolymers or exhibit cytotoxicity [7]. For example, glutaraldehyde, the most common crosslinking agent, presents handling difficulties and has contentious reports regarding the cytotoxicity of glutaraldehyde‐crosslinked materials [8]. Further problems related to the use of toxic crosslinkers are that the material needs to be washed and all hazardous components must be removed, as it could otherwise potentially be harmful during its application [9]. The investigation of more cell‐compatible and less toxic crosslinkers in combination with relevant biomaterials is crucial for developing products with suitable mechanical properties and adequate water stability for biomedical applications [10, 11]. Relevant research exists, for example, the combination of hyaluronic acid with 1,4‐butanediol diglycidyl ether (BDDE) and poly(propylene glycol) diglycidyl ether (PEGDE), shows excellent in vitro biocompatibility of the materials despite the detection of residual crosslinkers [12]. These crosslinking systems are not only investigated in research but are also implemented in approved medical devices such as dermal fillers, demonstrating their practical relevance for clinical applications.

In addition to potential toxicity caused by residual crosslinker molecules, alterations of the biomaterial itself—such as oxidation [13] or other chemical modifications, such as the introduction of glycoconjugates [14]—may influence the cytocompatibility and cell‐interaction at the application site. It is necessary to consider each case individually, taking into account the specific biomaterial and the product form in which it is processed. Furthermore, the chosen crosslinker and its composition, as well as the process parameters, will play a crucial role in the final product's biocompatibility.

Electrospinning is a versatile and efficient technique for producing nanoscale fibers from a variety of synthetic and natural materials, gaining traction in the biomedical field. Electrospun nonwoven mats are particularly promising due to their ability to mimic the extracellular matrix (ECM) and facilitate the delivery of active substances, thanks to their highly porous architecture and elevated surface‐to‐volume ratio [15, 16].

Electrospinning is the preferred technique for fabricating nonwoven mats, as their nanofibrous structure enhances cell adhesion, proliferation, and differentiation. Additionally, electrospinning enables the control of key parameters, including fiber diameter, surface morphology, porosity, mechanical properties, and fibrous architecture, allowing for the customization of nonwoven mats [17]. Electrospun collagen‐based nonwoven mats demonstrated favorable conditions for the adhesion, growth, and differentiation of human adipose‐derived mesenchymal stem cells with no cytotoxic effects observed, making them suitable substrates for regenerative applications [18]. Further examples of electrospun nonwoven mats and their cell interaction properties for tissue engineering and other biomedical applications are presented by Dadashpour and coworkers, and Olaret and coworkers [19, 20].

Especially interesting for the combination with more cell‐compatible crosslinkers are recombinantly produced biomaterials. For example, animal‐derived collagen can cause immunogenic reactions and have batch‐to‐batch variability. These problems can be avoided through recombinant production [21]. By combining compatible crosslinkers with recombinantly produced biomaterials, the risk of harmful effects for the user is minimized.

A new biomaterial with promising properties for cell‐interaction and drug delivery applications is VECOLLAN, a recombinant collagen‐like protein. VECOLLAN exhibits high biocompatibility, non‐inflammatory and non‐mutagenic properties, with no cytotoxicity, irritation, or sensitization observed in comprehensive in vitro and in vivo assays, supporting its potential as a safe biomaterial for long‐term medical applications [22]. In this work, VECOLLAN is abbreviated as “CLP”, the short form for “collagen‐like protein”.

In our previous work, we presented the optimized electrospinning parameters and a coaxial crosslinking approach to produce CLP‐based fibers with tunable water solubility. A detailed characterization of the swelling behavior and mechanical properties of the nonwoven mats was conducted. The experiments suggest that the nonwoven mats, due to their modifiable properties, could be promising candidates for applications in the biomedical field [23]. These results encouraged us to proceed, and therefore, the aims of the current studies are: 1) assessment of the cell compatibility of product candidates of our previous work, including their interaction and effects on cell viability and adhesion, 2) investigation of the chemical leachables from DMTMM crosslinked CLP‐fibers following ISO standards. The study investigates whether potentially unexpected by‐products can arise during electrospinning or crosslinking. Furthermore 3), spectroscopic investigations were conducted to examine the potential effects of crosslinking on the amide bands or other bands that could be caused by structural changes of the material.

## 2 Results and Discussion

### 2.1 Cytotoxicity

**FIGURE 1:**
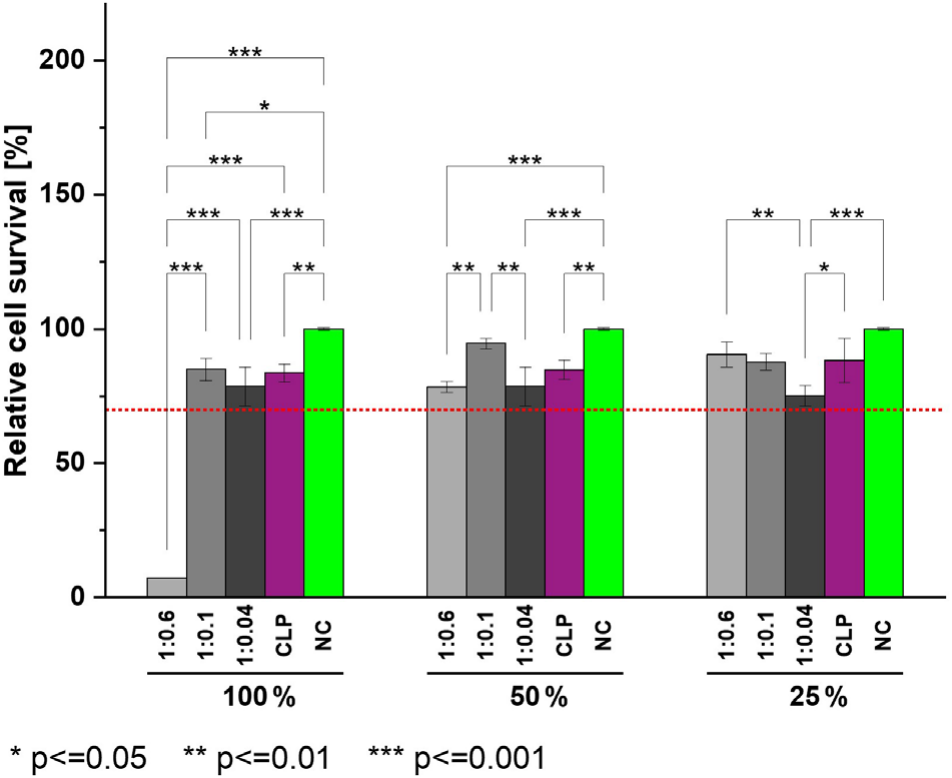
Cytotoxicity of CLP/PEO nonwoven mats crosslinked with DMTMM at molar ratios of CLP:DMTMM = 1:0.6, 1:0.1, and 1:0.04, as well as non‐crosslinked CLP, evaluated by MTS assay after extract preparation and incubation with cells. NC represents the negative control. The red dashed line at 70% indicates the cell viability threshold defined in ISO 10993–5. Horizontal brackets indicate statistically significant differences between groups (ANOVA followed by Tukey's post‐hoc test). Each extract was tested in triplicate (*n* = 3).

The absence of cytotoxicity is crucial for CLP‐based nonwoven mats in the biomedical sector [24, 25]. To analyze the effect of leachables, a cytotoxicity test was carried out, adapted from ISO standard 10993–5 [26]. This part of the ISO standard describes test procedures for the determination of in vitro cytotoxicity of medical devices. Here, it was used for the cytotoxicity evaluation of electrospun nonwoven mats [27, 28]. The cells were exposed to extracts of electrospun nonwoven mats at different dilutions (100%, 50%, 25%) and compared to a CLP solution. After 24 h exposition time, test solutions were replaced by MTS staining solution followed by absorption read‐out after a two‐hour reaction time (Figure 1). Following ISO standard 10993–5, the cell viability threshold is set to a minimum of 70% cell viability, which was surpassed in this experiment for all undiluted extracts except for the sample CLP:DMTMM 1:0.6. For this sample, after a 1:1 dilution, the cell viability was above the cytotoxicity threshold. The sample CLP:DMTMM 1:0.1 has the lowest crosslinker concentration at which no CLP release was seen in the dissolution test and showed good compatibility with the cells in the undiluted state. The cytocompatibility of CLP at a concentration of 43 mg/mL was confirmed. It is often essential to wash biomaterials thoroughly to eliminate any residual crosslinker molecules [29]. The viability of cells treated with extracts of a non‐soluble CLP nonwoven mat (CLP:DMTMM 1:0.1) and a partially soluble CLP nonwoven mat (CLP:DMTMM 1:0.04) was above the 70% threshold without washing them after production *via* electrospinning. With the coaxial crosslinking approach, we found an efficient way to produce electrospun nonwoven mats. Looking at the pairwise statistical comparison, all 100% extracts have a significant influence on the cell viability, compared to the negative control. Nevertheless, the findings of this experiment indicate that the residual crosslinker concentrations of CLP:DMTMM 1:0.1 and CLP:DMTMM 1:0.04 are low enough to be considered as non‐cytotoxic, suggesting that the washing step for its removal could be omitted in future processes to streamline manufacturing.

### 2.2 Cell Adhesion

**FIGURE 2:**
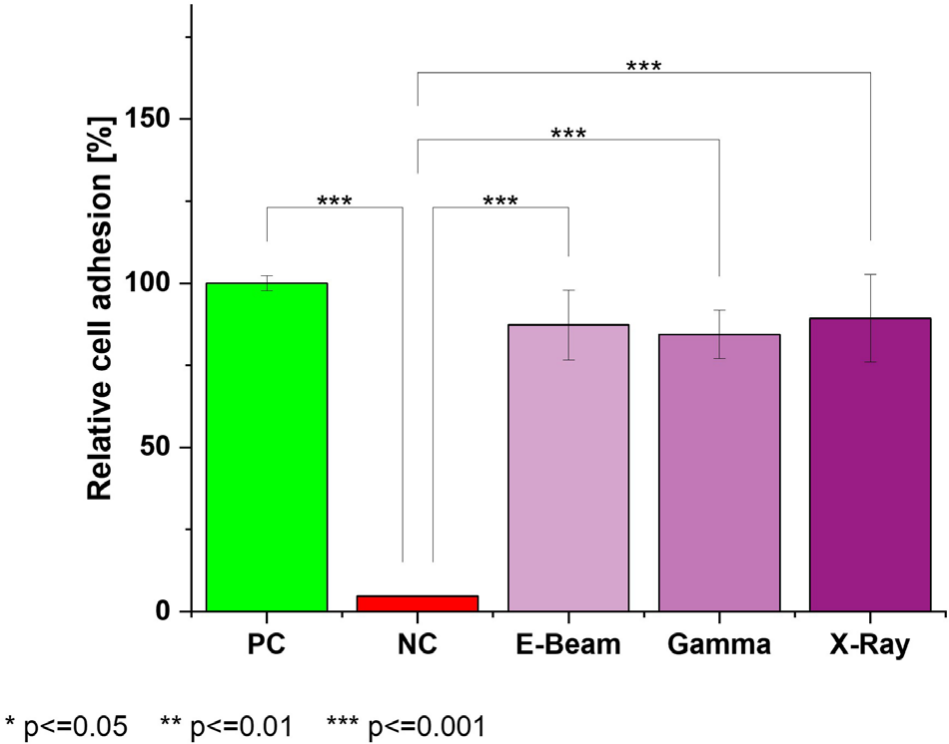
Relative NIH/3T3 adhesion on different surfaces. PC = positive control (plasma‐treated plate); NC = negative control (ultra‐low adhesion plate). Irradiated samples comprise CLP/PEO nonwoven mats stabilized by DMTMM crosslinked using a CLP:DMTMM MR of 1:0.1. Horizontal brackets indicate statistically significant differences between the respective groups (ANOVA, followed by Tukey's post‐hoc test). Each sample was tested in triplicate (*n* = 3).

Collagen is well known for its excellent cell‐adhesive properties [30, 31]. Both crosslinkers [32] and combinations of CLP with high proportions of PEO may result in reduced bioactivity [33, 34]. The nonwoven mats fabricated in this study contain only a small fraction of PEO. The efficiency of cell adhesion—or its absence—determines the potential application of the resulting product [35, 36, 37]. Crosslinked CLP/PEO mats were treated using gamma, e‐beam, and x‐ray irradiation before assessing the cell adhesion with NIH/3T3 cells. As shown in Figure 2, all treated samples exhibited relatively good cell adhesion, ranging from 84%–89%. Data was normalized to the average signal of the positive control (plasma‐treated cell culture surface). The irradiation method had no significant influence on the adhesion properties of the nonwoven mats, and there is no significant difference between the nonwoven mats and the positive control (analysis of variance, followed by Tukey's post‐hoc test, 95% confidence interval). In summary, a comparable cellular attachment was observed for all test specimens, and the material was evaluated as cell adhesive for the tested murine fibroblasts.

### 2.3 Leachables Test

#### 2.3.1 LC‐MS Analysis Oriented Toward ISO 10993‐18

The ISO standard 10993‐18 describes a framework for identifying biological hazards and for estimating and controlling the biological risks potentially caused by component materials, using a step‐by‐step approach to characterize a medical device. In this experiment, we investigated the release of chemical substances (extractables). The calculation of the analytical extraction threshold (AET) was omitted at this early stage of development. The experiment provides a qualitative overview of the chemical extractables of the nonwovens.

**FIGURE 3:**
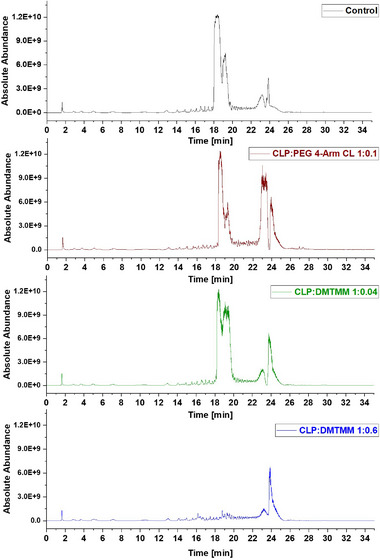
Comparison of the LC‐MS‐Chromatograms (TIC, m/z 300 – 2000) obtained from the analysis of the “Control” (black) and the samples “CLP:PEG 4‐Arm CL 1:0.1” (brown), “CLP:DMTMM 1:0.04” (green), and “CLP:DMTMM 1:0.6” (blue).

**FIGURE 4:**
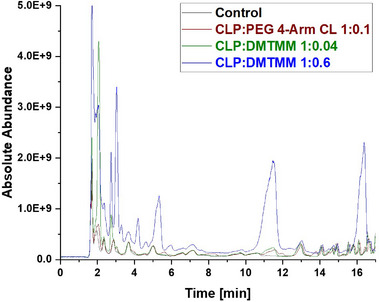
Zoom (RT = 0 – 17 min) into the overlay of the LCMS‐Chromatograms (TIC, m/z 100 – 1000) obtained from the analysis of the Control sample (black) and the samples PEG 4‐Arm CL 1:0.1 (brown), CLP:DMTMM 1:0.04 (green), and CLP:DMTMM 0.6 (blue).

The extracts from the nonwoven mats CLP:PEG 4‐Arm CL 1:0.1, CLP:DMTMM 1:0.04, and CLP:DMTMM 1:0.6 were analyzed via Liquid Chromatography‐Mass Spectrometry (LC‐MS, Figure 3 and Figure 4). All samples were prepared without a washing step before the extraction process. In case of the CLP:DMTMM 1:0.6 sample, unexpected side products were found that were not based on the theoretical reaction mechanism of the crosslinker. The sample with a CLP:DMTMM ratio of 1:0.04 exhibited fewer peaks corresponding to crosslinker reaction products compared to the sample with a ratio of 1:0.6. Since the peaks of sample CLP:DMTMM 1:0.04 were considerably weaker or not visible at all compared to sample CLP:DMTMM 1:0.6, only the latter will be interpreted in detail below. The samples CLP:PEG 4‐Arm CL and Control showed no unexpected side products or unknown impurities.

In the chromatographic region between retention time (RT) 18.32 and 19.22 min (Figure 3), peaks corresponding to CLP were observed. As expected, all samples except for CLP:DMTMM 1:0.6 exhibited these peaks. This absence in sample CLP:DMTMM 1:0.6 suggests that the crosslinker concentration was sufficiently high to fully crosslink CLP, thus preventing its release. This result supports the findings of the dissolution tests that were conducted in our previous work [23].

Furthermore, at retention times of approximately 23.07 and RT 23.73 min (Figure 3), all samples displayed peaks corresponding to PEO species. These were identified within the molecular weight (MW) ranges of 1500–2000 Da and 350–1000 Da, respectively. Although the PEO used was specified to have a molecular weight of 400 kDa, the presence of fragments within these MW ranges was anticipated. It was not possible to detect high‐molecular‐weight PEOs in the 400 kDa range using this method.

Despite the potential for DMTMM to react with the OH‐terminus of PEO, the concentration in the CLP:DMTMM 1:0.6 sample was not high enough to crosslink all PEO chains.

**FIGURE 5:**
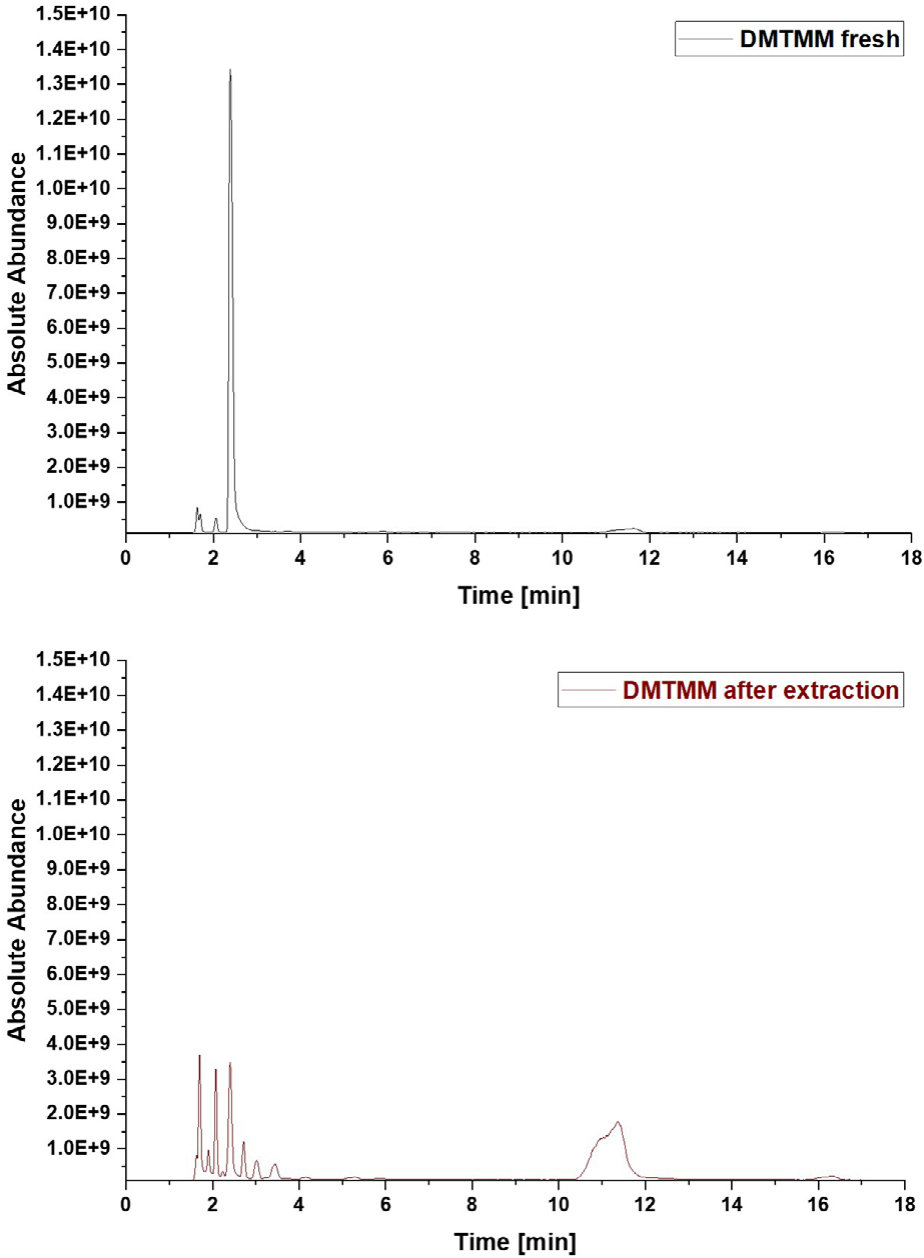
Comparison of the LC‐MS‐Chromatograms (TIC, m/z 100 – 1000) obtained from the analysis of a freshly prepared solution (top) and an extracted solution (bottom) containing pure DMTMM cross‐linker. The injection volume differs from the generally applied method: 2 µL instead of 25 µL.

The CLP:DMTMM 1:0.6 sample shows a prominent peak at a retention time of 16.32 min corresponding to a mass‐to‐charge ratio (m/z) of 297.10 (Figure 4). The molecule at this retention time is 2,2′‐oxybis[4,6‐dimethoxy‐1,3,5‐triazine] (Table 1). To investigate whether 2,2′‐oxybis[4,6‐dimethoxy‐1,3,5‐triazine] arises during coaxial electrospinning, is already present in the original chemical, or arises during the extraction process, further experiments were carried out. Figure 5 (top) shows the chromatogram of a freshly prepared DMTMM solution. Figure 5 (bottom) shows the chromatogram of DMTMM that has gone through the complete extraction process. At a retention time of 16.32 min, (m/z) 297.10, the freshly prepared DMTMM solution shows a barely detectable peak that is slightly enlarged by the extraction process.

**TABLE 1:** Accurate mass data, calculated formulas, and proposed structures.

RT, MS [min]	Ion m/z	Calculated formula	Proposed structure
	116.11	C6 H14 O N ΔM 1.03 ppm	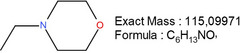 , as [M+H]^+^
	102.09	C5 H12 O N ΔM 1.86 ppm	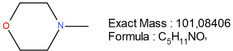 , as [M+H]^+^
1.70	130.03	C3 H4 O3 N3 ΔM 0.02 ppm	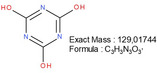 , as [M+H]^+^
	144.04	C4 H6 O3 N3 ΔM 1.86 ppm	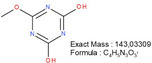 , as [M+H]^+^
2.09	158.06	C5 H8 O3 N3 ΔM – 1.76 ppm	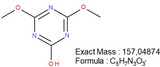 , as [M+H]^+^
2.14	116.03	C4 H6 O3 N ΔM 1.04 ppm	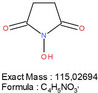 , as [M+H]^+^ Very low response, almost not visible in TIC‐data. Molecular structure indicates the difficulty in ionizing the molecule.
2.75	172.07	C6 H10 O3 N3 ΔM – 0.80 ppm	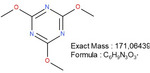 , as [M+H]^+^
3.04	177.05	C5 H9 O5 N2 ΔM – 0.16 ppm	Unknown, as [M+H]^+^
4.15	186.09	C7 H12 O3 N3 ΔM – 0.96 ppm	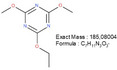 , as [M+H]^+^
5.32	191.07	C6 H11 O5 N2 ΔM – 1.09 ppm	Unknown, as [M+H]^+^
11.39	227.11	C7 H13 O2 N7 ΔM – 1.48 ppm	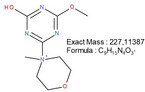 , as [M]^+^
16.32	297.09	C10 H13 O5 N6 ΔM – 1.53 ppm	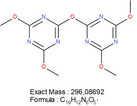 , as [M+H]^+^
18.32	—	—	Protein with a molecular weight of approximately 22 – 23 kDa **Hypothesis**: CLP/CLP‐breakdown product
19.22	—	—	Protein with a molecular weight of approximately 22 – 23 kDa **Hypothesis**: CLP/CLP‐breakdown product
23.07	—	—	PEO MW ≈ 1500 – 2000 Probably a minor pollution of the used PEO 400 kDa PEO 400 kDa itself is too heavy/large to be detected by LCMS.
23.39	225.20	C13 H25 O N2 ΔM – 0.09 ppm	Unknown, as [M+H]^+^
23.73	—	—	PEO MW ≈ 350 – 1000 Probably a minor pollution of the used PEO 400 kDa. PEO 400 kDa itself is too large to be detected by this LCMS method. The later elution of this PEO compared to the PEO eluting at RT = 23.07 min might indicate a nonpolar group attached to the PEO.

The peak at a RT of 11.39 (m/z) 227.11, Figure 5 (bottom), shows a molecule that seems to arise largely from DMTMM in both the electrospinning and extraction processes. This molecule is already present in small amounts in the original DMTMM substance.

The molecule with an RT of 4.15, (m/z) 186.09, was detected in the CLP:DMTMM 1:0.6 sample (Figure 4) and the pure DMTMM sample only after the extraction process (Figure 5, bottom). Since the extraction agent contains ethanol, the reaction that leads to the formation of this molecule may occur during the extraction process.

The expected leaving groups of DMTMM during the crosslinking reaction(N‐Methylmorpholine and 4,6‐Dimethoxy‐1,3,5‐triazin 2‐ol) are shown in Table 1 at a RT of 1.70, (m/z) 102.09, and at a RT of 2.09, (m/z) 158.06. These molecules are found in both the CLP:DMTMM 1:0.04 and the CLP:DMTMM 1:0.6 sample.

However, there are many other peaks in this RT range (Figure 4), which is why an exact differentiation is difficult. Interestingly, both molecules (RT 1.70 and RT 2.09) are also found in the original substance (Figure 5, top) and formed during pure DMTMM substance extraction. Therefore, after extraction, it was not possible to assess how much of the added crosslinker had actually reacted during the coaxial crosslinking process and how much only reacted during the extraction process.

Generally, it can be said that the extraction process completely alters the spectrum of the pure DMTMM substance. All molecules found in the CLP:DMTMM 1:0.6 sample after extraction were also found in the extracted pure substance of DMTMM. Therefore, the electrospinning process itself does not appear to produce any side product reactions.

To find extractable molecules of a nonwoven mat, crosslinked with the PEG 4‐Arm CL, the sample CLP:PEG 4‐Arm CL 1:0.1 was investigated. The leaving group formed upon successful crosslinking of the PEG 4‐Arm CL is N‐Hydroxysuccinimide. At a retention time of 2.14, (m/z) 116.03, N‐Hydroxysuccinimide could be detected with a very low signal. A possible explanation is that the molecule is difficult to ionize due to its structure. No other chemical compounds originating from this crosslinker were found.

An attempt was made to quantify individual molecules using quantitative nuclear magnetic resonance (QNMR) spectroscopy. Despite employing this highly sensitive analytical technique, a quantification of individual molecules was not possible.

### 2.4 FTIR Analysis

Various samples of the individual substances and the products with and without crosslinker were investigated. The exact composition of the examined samples is listed in Table 2. In comparison to the Control sample (CLP + PEO 400 kDa), the nonwoven mats containing additional DMTMM (CLP:DMTMM 1:0.6) exhibited shifts in band positions at several spectral regions, along with faint indications of an additional absorption in the carbonyl region. These observations may be caused by crosslinking. However, no quantitative conclusions can be drawn regarding the efficiency of crosslinking.

**TABLE 2:** Investigated samples in the FTIR‐spectrometer.

**Sample name**	**Composition**
CLP	Pure CLP
Control	CLP + PEO 400 kDa (no crosslinker)
DMTMM	Pure DMTMM
PEO 400 kDa	PEO 400 kDa
CLP:DMTMM 1:0.6	CLP + PEO 400 kDa + DMTMM MR 1:0.6
CLP:PEG 4‐Arm CL 1:0.1	CLP + PEO 400 kDa + PEG 4‐Arm CL MR 1:0.1

The process of crosslinking CLP with DMTMM results in the formation of new amide bonds [38]. Consequently, an investigation was conducted to determine whether any changes in the amide bands could be observed in the FTIR spectrum.

**FIGURE 6:**
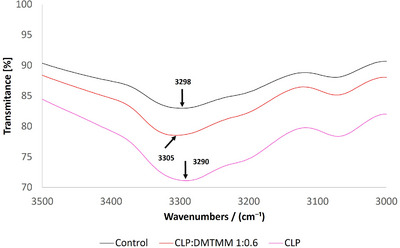
FTIR spectra in the N–H stretching vibration region (3500–3000 cm^−^^1^) for the Control sample, the CLP:DMTMM 1:0.6 sample, and the CLP sample.

Slight shifts in the absorption of the amide A band at approximately 3300 wave numbers (cm^−1^) may indicate a change in structure (Figure 6). An interaction due to different residual moisture levels in the samples could also contribute to a shift in the absorption maxima.

The COO‐vibration of the amino side chain (Table 3) was assigned according to the suggestion of Tamaddon and coworkers [39]. A shift in the band position between the Control sample and the CLP:DMTMM 1:0.6 sample by approximately 5 wavenumbers was found (Figure 6). The vibration of the PEO also shows a shift in the band position between the PEO 400 kDa sample and the CLP:DMTMM 1:0.6 sample. However, this is already detectable in the Control sample and may be due to altered interaction caused by mixing the PEO 400 kDa with the CLP rather than crosslinking of the CLP. This shift could be caused by hydrogen bonding between the C–O groups of PEO and the N–H groups of the CLP [40].

**TABLE 3:** CH‐deformation and COO‐vibration in wave numbers (cm^−^^1^).

**Band**	**CLP**	**Control sample**	**CLP:DMTMM 1:0.6**	**PEO**
CH‐deformation	1448	1451	1450	1455
COO	1397	1397	1402	—

To identify potential changes in amid bands resulting from the crosslinking process, the alterations observed during collagen crosslinking with glutaraldehyde in the study by Chang & Tananka [41] were taken into consideration. Chang & Tanaka associated the bands of wave numbers 1685 and 1653 cm^−1^ with collagen crosslinking. However, the differences described in their work due to the crosslinking of collagen are very close to water vapor bands and appear to correlate to some extent with the intensity of water vapor, so the comparison was discontinued.

To investigate if the bands presented in the study by Chang & Tanaka, comparative measurements of two 100% transmission lines (measurements of the empty transmission sample chamber) from different measurement days were subtracted from each other in such a way that the best possible spectrum of water vapor was obtained. This spectrum (not presented in this publication) was amplified in intensity by multiplication factors of 1, 3, and 20 and added to the average spectrum of the three CLP measurements. In the spectrum with the most intense water vapor bands, bands are visible at approximately 1684 and 1653 wave numbers. The published FTIR spectrum by Chang & Tanaka is very likely to be affected by water vapor absorption. Additional FTIR spectra showing slight band changes in the region of peak absorption of the DMTMM molecule (Figure S1) and shifts in the Amide I and Amide II bands (Figure S2) are presented in the supporting material.

For the sample CLP:PEG 4‐Arm CL 1:0.1, the spectra are highly compatible with the mixture of spectra of the Control sample and the spectrum of the pure PEG 4‐Arm CL (spectrum not shown). There are no significant differences that could be interpreted as evidence of crosslinking. The amount of crosslinking agent used may be too low to be detected by FTIR spectroscopy.

## 3 Conclusions

In conclusion, this study provides the first steps in investigating the cytocompatibility and cell interactions of electrospun VECOLLAN‐based product candidates, demonstrating that the majority of samples maintained cell viability above the ISO standard 10993–5 cell viability threshold, with the CLP:DMTMM 1:0.1 sample showing good cytocompatibility and effective crosslinking without CLP dissolution when exposed to water. The nonwoven mats showed good cell adhesion, relevant for application in the tissue‐engineering sector, regenerative medicine, or drug delivery. Although the coaxial crosslinking method proved to be efficient, the chemical leachables evaluation of the nonwoven mats showed some impurities, which were already present in the raw material DMTMM. This proves that the manufacturing processes do not harm the chemical profile of the final product. For clinical manufacturing, the usage of a well‐characterized raw material is therefore crucial. The FTIR spectroscopic investigation provides weak band shifts at different wavenumbers, which could be caused by crosslinking. The attempt to establish a correlation between the amount of crosslinker used and band intensity in the spectrum has failed. In addition to investigating other crosslinkers and crosslinking methods, future research on VECOLLAN‐based nonwoven mats should focus on additional cell types to obtain a more comprehensive understanding of their biocompatibility and potential fields of application.

## 4 Experimental Section/Methods

### 4.1 Graphical Abstract

The graphical abstract was created using BioRender (www.biorender.com).

### 4.2 Use of the Word “Fibers”

In some experiments, instead of individual fibers, a large number of stacked fibers forming a nonwoven mat was used. Therefore, when referring to fibers, it may sometimes involve numerous fibers forming a nonwoven mat.

### 4.3 Materials

VECOLLAN from Evonik Industries AG, Germany. Polyethyleneoxide (PEO) with a molecular weight of 400 kDa from Merck KGaA, Germany. 4‐(4,6‐Dimethoxy‐1,3,5‐triazin‐2‐yl)‐4‐methylmorpholinium Chloride (DMTMM) from TCI Deutschland GmbH, Germany. The crosslinker (CL) 4‐Arm‐polyethylene glycol‐succinimidylglutarate‐ester (PEG 4‐Arm CL) with a molecular weight of 10 kDa (PEG 4‐Arm CL) from JenKem Technology USA Inc. Acetone EMSURE (Merck KGaA, Germany). 3‐(4,5‐dimethylthiazol‐2‐yl)‐2,5‐diphenyltetrazolium bromide (MTS) from Merck KGaA, Germany.

### 4.4 Electrospinning

Electrospinning was conducted using a FLUIDNATEK LE‐50 electrospinning equipment (BIOINICIA FLUIDNATEK SLU, Spain). The solvent mixture for all spinning formulations comprised double‐distilled water/acetone (9:1 (v/v)). The fibers were produced with or without crosslinkers, depending on the experiment. The applied voltage to generate the fibers was 14–22 kV. The negative voltage on the flat collector was −5 kV. The temperature in the climate chamber of the electrospinner was set to 25°C and the relative humidity to 40% for all experiments. The spinneret to collector distance was 22.5 cm, and the fibers were collected on aluminum foil or baking paper on the collector. Each formulation was loaded into a 5 mL polypropylene syringe purchased from B.Braun SE, Germany. For monoaxial electrospinning, the spinning formulations were pumped through a VIEWEG GmbH (Germany) dosing needle with an inner diameter of 0.41 mm and a capillary length of 12.7 mm. For coaxial electrospinning, the spinning formulations were pumped through a VIEWEG GmbH (Germany) dosing needle with an inner diameter of 1.19 mm. The inner needle of the coaxial spinning head (BIOINICIA FLUIDNATEK SLU, Spain) had an inner diameter of 0.6 mm and an outer diameter of 0.9 mm. For the coaxial electrospinning, the shell phase flow rate was 600 µL h^−1^ and the core phase, 110 µL h^−1^. The crosslinker concentrations of the spinning formulations and the molar ratios of reactive CLP groups to reactive crosslinker groups are listed in Table 4.

**TABLE 4:** Composition and cross‐linker concentration of various formulations.

**CLP: Crosslinker molar ratio**	**Cross‐linker sol. conc. [mg/mL]**	**Molar ratio (CLP:Cross‐linker)**
CLP:DMTMM 1:0.6	173.2	1:0.6
CLP:DMTMM 1:0.1	28.9	1:0.1
CLP:DMTMM 1:0.04	11.5	1:0.04
CLP:PEG 4‐Arm CL 1:0.1	164.8	1:0.1
Control (no Cross‐linker)	/	/

### 4.5 Calculation of the Molar Ratio Between CLP and Crosslinker

For DMTMM, the ratio of reactive groups of CLP to a molecule of crosslinker refers to the number of carboxylic acid groups of one molecule of CLP. One molecule of DMTMM has one reactive site that can undergo crosslinking reactions. Therefore, a ratio of reactive groups to DMTMM of 1:1 corresponds to one molecule of CLP to 36 molecules of DMTMM.

For the PEG 4‐Arm CL, the ratio of reactive groups of CLP to a molecule of crosslinker refers to the number of primary amines of one molecule of CLP. One molecule of PEG 4‐Arm CL has four reactive sites that can undergo crosslinking reactions. Therefore, a ratio of reactive CLP groups to PEG 4‐Arm of 1:1 corresponds to one molecule of CLP to 5.75 molecules of PEG 4‐Arm CL.

### 4.6 Cytotoxicity

The CellTiter 96 Aqueous Non‐Radioactive Cell Proliferation Assay (Promega GmbH, Germany) measures the metabolic activity of cells by quantifying the concentration of reduction equivalents (NADH & NADPH) produced by viable cells during glycolysis. This was achieved by reducing the 3‐(4,5‐dimethylthiazol‐2‐yl)‐2,5‐diphenyltetrazolium bromide (MTS) reagent to a measurable purple formazan product, which serves as an indirect indicator of cell viability. The assay follows the manufacturer's protocol and is oriented toward ISO standard 10993–5. Briefly, cells (NIH/3T3, DSMZ‐Deutsche Sammlung von Mikroorganismen und Zellkulturen GmbH, Germany) were seeded at 1 × 10^4^ cells per well (1 × 10^5^ cells mL^−1^, 0.1 mL) in a transparent 96‐well plate. Samples were extracted (50 mg of nonwoven per mL cell culture medium) in cell culture medium for 48 h, 37°C, 5% CO_2_. Undiluted (100%), 1:1 diluted (50%), and 1:4 diluted (25%) extracts from nonwoven mats were prepared. As control, a 43 mg mL^−1^ CLP solution in culture medium was used for equal CLP content comparable to the non‐crosslinked CLP/PEO nonwoven. After 24 h of incubation under culture conditions, the cell medium was replaced with 100 µL of the test solutions.

For the positive control, 100 µl of 1% SDS in cell culture medium was used. For background control, the complete culture medium serves as a negative control. Cells were exposed to the extracts for 24 h under cell culture conditions. Then, extracts were replaced by 100 µl fresh culture medium and 20 µl of premixed MTS reagent solution per well (prepared according to the user manual). The test plate was incubated for 2 h (37°C, 5% CO_2_). Absorbance was detected at 490 nm using a multi‐plate reader (Tecan Infinite Pro 200, Tecan Group Ltd., Switzerland). The background signal was subtracted from each test sample, followed by normalization to the negative control. The sample mean and standard deviation were calculated.

### 4.7 Cell Adhesion

To evaluate cell adhesion, mouse fibroblasts (NIH/3T3) were seeded onto various electrospun nonwoven mats. Composite mats consisting of collagen‐like protein (CLP) and polyethylene oxide (PEO) were fabricated and crosslinked using DMTMM at a molar ratio of CLP to DMTMM of 1:0.1. Circular samples (Ø 15 mm) were punched out and treated with radiation to reduce biological burden using gamma irradiation (17.56–18.85 kGy), electron beam (17.61–19.59 kGy), or x‐ray (19.3–20.6 kGy). Samples were placed into sterile, ultra‐low adhesive 12‐well microtiter plates (Costar, flat bottom, polystyrene; Corning Inc., USA, Cat. No. 3737). To prevent sample displacement during incubation, autoclaved 10 mm stainless steel Swagelok rings (Swagelok Company, USA, Cat. No. SS‐10M4‐1) were placed on top of each nonwoven mat. Each well was seeded with 3 × 10^5^ NIH/3T3 cells suspended in 2 mL of DMEM medium (Gibco, Cat. No. 10313‐021), supplemented with 15% FBS and GlutaMAX (Gibco, Cat. No.: 35050061). To mitigate potential microbial contamination, gentamycin (30 µg mL^−1^) was added.

3 × 10^5^ NIH/3T3 cells suspended in 2 mL of DMEM medium in plasma‐treated plates (Greiner BioOne; Cat. No. 665180) served as a positive control for signal normalization. After 24 h incubation at 37°C, 5% CO_2_, and humidified conditions, wells were washed twice with 1×PBS and replenished with 1 mL of a 1 mg mL^−1^ MTT solution in DMEM medium. Plates were incubated for 4 h at 37°C. Then, the medium was replaced by 1 mL of DMSO. Plates were shaken for 15 min at 300 rpm on an orbital shaker. From each well, 3 × 200 µL aliquots were transferred into 96‐well plates (Greiner BioOne, Cat. No. 650161). Absorbance was measured at 570 nm using a microplate reader (Spark, Tecan Group Ltd., Switzerland). 1 mg mL^−1^ MTT in DMEM medium was used as a background control. Data was processed in Microsoft Excel by subtracting the mean background signal and normalizing to the average positive control, expressed as percentage adhesion. Data visualization was performed using OriginPro 2022.

For more accurate signal interpretation, it must be considered that the nonwoven mat diameter was 18 mm, which did not fully cover the well surface (22 mm). The positive control formed a confluent cell layer across the entire well area, resulting in a higher overall signal. To correct for this, we conducted a normalization per scaffold area for the investigated nonwovens. The normalization was also conducted for the positive and negative controls.

### 4.8 Chemical Leachables

The LC‐MS analysis was performed using a Vanquish UHPLC system coupled with an Orbitrap Q Exactive HF mass spectrometer (Thermo Scientific, Life Technologies GmbH, Germany). The experiment was conducted in positive electrospray ionization (ESI) mode. The mass spectrometer was operated with a mass range of m/z 100 – 1000 u and m/z 300 – 2000 u, at a resolution of 60.000.

Chromatographic separation was achieved using a 150 × 4.6 mm Zorbax SB300 C8 column (K107). The mobile phase consisted of Eluent A (water with 0.1% trifluoroacetic acid) and Eluent B (acetonitrile/water, 9/1, v/v, with 0.1% trifluoroacetic acid). The gradient elution was programmed as follows: start with 9% Eluent B for 8 min, then increase to 29% Eluent B over 12 min, followed by a ramp to 100% Eluent B in 5 min. The flow rate was maintained at 1.0 mL min^−1^, with the column temperature set to 35°C. The autosampler was kept at 10°C, and an injection volume of 25 µL was used for each sample.

#### 4.8.1 Sample Preparation

To extract potential leachables and extractables, an ethanol (EtOH) and sodium chloride (NaCl) solution [0.9% (w/w)] was prepared at a 2:3 (v/v) ratio. A 50 mg sample of the material was submerged in 1 mL of this mixture. The sample containers were sealed with parafilm and incubated for 48 ± 2 h at a temperature of 37 ± 1°C. Following incubation, the samples were centrifuged for 10 min at 15 000 rpm. The resulting clear supernatant was filtered using a 0.22 µm PVDF filter. The filtered eluate was then used for subsequent analysis.

### 4.9 FTIR Spectroscopy

Fourier Transform Infrared Spectroscopy (FTIR) was conducted with an FTIR spectrometer iS50 (Thermo Fisher Scientific Inc., USA) in triplicate. Mean spectra were calculated to compare the band positions. The scanning range was 400–4000 cm^−1^, and the resolution was set at 4 cm^−1^. All measurements were executed on materials sourced from the same sample vessel to ensure consistency. The crosslinkers and polyethylene glycol PEO samples were each measured once for comparative analysis. The experiments utilized Attenuated Total Reflectance (ATR) mode, employing a diamond crystal as the measuring medium.

## Funding

This study was funded by internal funds of the Evonik Operations GmbH.

## Conflicts of Interest

Christoph Krauss reports that financial support was provided by Evonik Operations GmbH. Maria Montero Mirabet reports that financial support was provided by Evonik Operations GmbH. Christoph Krauss reports a relationship with Evonik Operations GmbH that includes: employment and equity or stocks. Maria Montero Mirabet reports a relationship with Evonik Operations GmbH that includes: employment and equity or stocks. Blerta Sonja Thaqi reports a relationship with Evonik Operations GmbH that includes: employment and equity or stocks. Sven Weber reports a relationship with Evonik Operations GmbH that includes: employment and equity or stocks. Christoph Krauss has a patent pending for Evonik. Maria Montero Mirabet has a patent pending for Evonik. Karsten Mäder declares that he has no known competing financial interests or personal relationships that could have appeared to influence the work reported in this paper.

## Supporting information




**Supporting File**: mabi70180‐sup‐0001‐SuppMat.docx.

## Data Availability

The data that support the findings of this study are available from the corresponding author upon reasonable request.
