# Supplementary material for: Animal‐Free Derived Collagen‐Like Protein Based Electrospun Nanofibers for Biomedical Applications: Cell Interactions Studies
Source: Macromol Biosci. 2026 Apr 9;26:e70180. doi: 10.1002/mabi.70180 (PMC13064797; doi:10.1002/mabi.70180)
Supplement: Supplementary file 1 — Supporting File: mabi70180‐sup‐0001‐SuppMat.docx. [file MABI-26-e70180-s001.docx]

Supplementary information

To find hints of crosslinking, additional FTIR bands were analyzed in more detail.

At 1374 cm^-1^, a weak hint of a band is observed in the CLP:DMTMM 1:0.6 sample, while no absorption is detected in the Control sample in this region (Figure S1). The DMTMM molecule exhibits a peak absorption around 1364 wavenumbers. Vibrations in this region could be attributed to the N₂C₃ stretching combined with C₃H and C₅H bending of the triazine ring [1]. A derivative of DMTMM may be present; however, the absorption is too weak for a definitive identification.


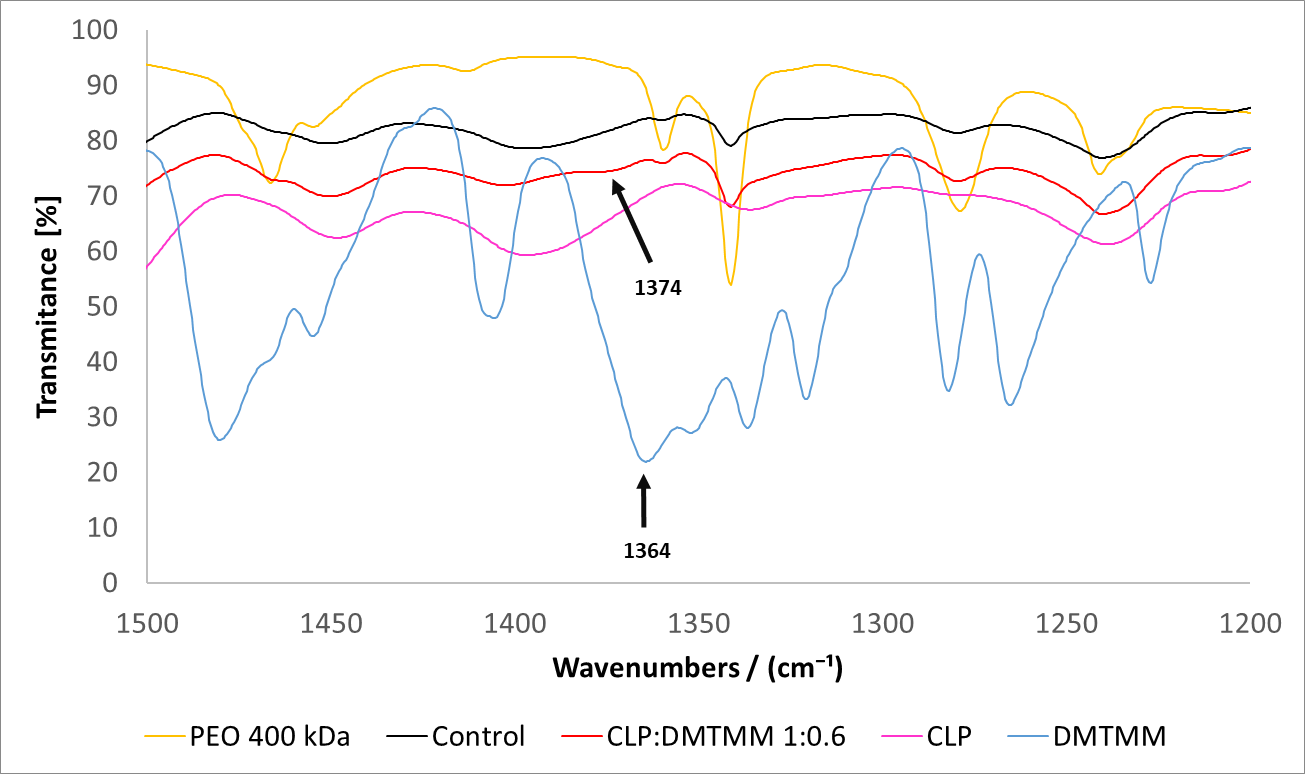


Figure S1: IR spectra in the area of the CH-deformation and the COO-vibration region (1500-1200 cm⁻¹) for PEO 400 kDa sample, the Control sample, the CLP:DMTMM 1:0.6 sample, the CLP sample, and the pure DMTMM sample.

A semi-quantitative comparison of the vibrations of amide groups to the PEO vibrations was performed, but no differences were detected. Given that CLP already contains numerous amide groups, the relative increase in amide bonds resulting from crosslinking is likely too minor to be detected through FTIR spectroscopy.

The amide I and amide II bands display a shift in their absorption maxima, as indicated in Table S1. The difference observed between the pure CLP spectrum and the Control sample is greater than that between the Control sample and the CLP:DMTMM 1:0.6 sample (Figure S2). This variation may be linked to changes in interactions due to structural modifications of the amide groups or the formation of new amide groups, which absorb and overlap at slightly shifted band positions.

Table S1: Amide I and Amide II bands of samples in wavenumbers (cm­^-1^).

| **Band** | **CLP** | **Control** | **CLP:DMTMM 1:0.6** |
| --- | --- | --- | --- |
| Amide I | 1643 | 1650 | 1651 |
| Amide II | 1537 | 1543 | 1548 |


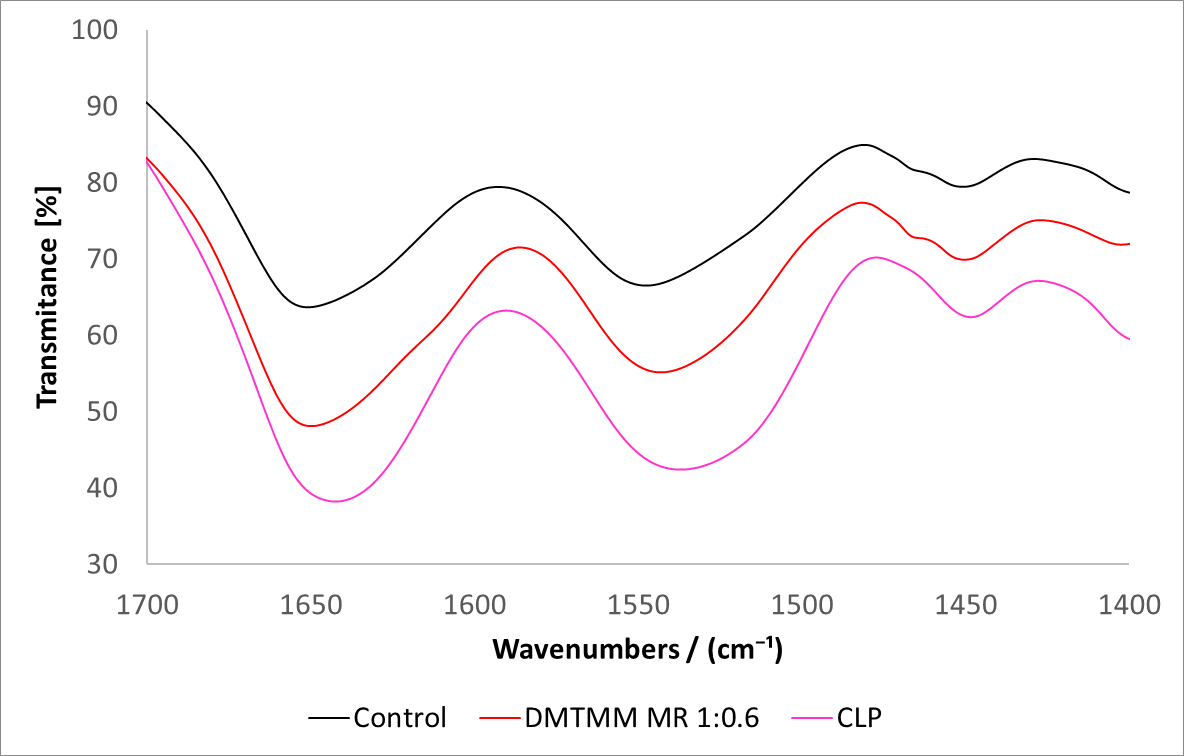


Figure S2: Comparison of FTIR spectra in the N–H stretching vibration region (1700–1400 cm⁻¹) for the Control sample, the CLP:DMTMM 1:0.6 sample, and the CLP sample.

References

[1] D.T. Bach, F. Hegelund, B.J. A., N.F. M., P.M. H., The High-Resolution Infrared Spectrum of 1,2,4-Triazine Vapor between 550 and 1700 cm-1, Journal of Molecular Spectroscopy 198 (1999) 77–93.
